# Supplementary material for: The brain reacting to COVID-19: analysis of the cerebrospinal fluid proteome, RNA and inflammation
Source: J Neuroinflammation. 2023 Feb 9;20:30. doi: 10.1186/s12974-023-02711-2 (PMC9909638; doi:10.1186/s12974-023-02711-2)
Supplement: Supplementary file 1 — Additional file 1. Supplementary tables and figures. [file 12974_2023_2711_MOESM1_ESM.pdf]

Supplementary material

Supplementary Table 1: Protein Abbreviation List for Figure 1

Protein.Names	First.Protein.Description
VGF_HUMAN	Neurosecretory protein VGF
TYRO3_HUMAN	Tyrosine-protein kinase receptor TYRO3
TIMP2_HUMAN	Metalloproteinase inhibitor 2
TIMP1_HUMAN	Metalloproteinase inhibitor 1
TICN1_HUMAN	Testican-1
SPRL1_HUMAN	SPARC-like protein 1
SPRC_HUMAN	SPARC
SLIK1_HUMAN	SLIT and NTRK-like protein 1
SHPS1_HUMAN	Tyrosine-protein phosphatase non-receptor type substrate 1
SGCE_HUMAN	Epsilon-sarcoglycan
SE6L2_HUMAN	Seizure 6-like protein 2
IC1_HUMAN	Plasma protease C1 inhibitor
A2AP_HUMAN	Alpha-2-antiplasmin
AACT_HUMAN	Alpha-1-antichymotrypsin
A1AT_HUMAN	Alpha-1-antitrypsin
SEM7A_HUMAN	Semaphorin-7A
LYAM1_HUMAN	L-selectin
7B2_HUMAN	Neuroendocrine protein 7B2
SCG2_HUMAN	Secretogranin-2
RNAS4_HUMAN	Ribonuclease 4
RNAS1_HUMAN	Ribonuclease pancreatic
RGMB_HUMAN	RGM domain family member B
RET4_HUMAN	Retinol-binding protein 4
EPCR_HUMAN	Endothelial protein C receptor
PRIO_HUMAN	Major prion protein
KPYM_HUMAN	Pyruvate kinase PKM
PGRP2_HUMAN	N-acetylmuramoyl-L-alanine amidase
PEBP4_HUMAN	Phosphatidylethanolamine-binding protein 4
PCSK1_HUMAN	ProSAAS
A1AG2_HUMAN	Alpha-1-acid glycoprotein 2
A1AG1_HUMAN	Alpha-1-acid glycoprotein 1
OMGP_HUMAN	Oligodendrocyte-myelin glycoprotein
NRX2A_HUMAN	Neurexin-2
NRCAM_HUMAN	Neuronal cell adhesion molecule
NPTXR_HUMAN	Neuronal pentraxin receptor
NPTX1_HUMAN	Neuronal pentraxin-1
NEGR1_HUMAN	Neuronal growth regulator 1
LYSC_HUMAN	Lysozyme C
LYVE1_HUMAN	Lymphatic vessel endothelial hyaluronic acid receptor 1
LYNX1_HUMAN	Ly-6/neurotoxin-like protein 1
LY6H_HUMAN	Lymphocyte antigen 6H
A2GL_HUMAN	Leucine-rich alpha-2-glycoprotein
LAMP2_HUMAN	Lysosome-associated membrane glycoprotein 2
K2C1_HUMAN	Keratin, type II cytoskeletal 1
KLKB1_HUMAN	Plasma kallikrein

KLK6_HUMAN	Kallikrein-6
IGJ_HUMAN	Immunoglobulin J chain
ITIH4_HUMAN	Inter-alpha-trypsin inhibitor heavy chain H4
ITIH2_HUMAN	Inter-alpha-trypsin inhibitor heavy chain H2
ITIH1_HUMAN	Inter-alpha-trypsin inhibitor heavy chain H1
LV319_HUMAN	Immunoglobulin lambda variable 3-19
LV147_HUMAN	Immunoglobulin lambda variable 1-47
IGLL5_HUMAN	Immunoglobulin lambda-like polypeptide 5
IGLC3_HUMAN	Immunoglobulin lambda constant 3
KV401_HUMAN	Immunoglobulin kappa variable 4-1
KV320_HUMAN	Immunoglobulin kappa variable 3-20
KV105_HUMAN	Immunoglobulin kappa variable 1-5
IGKC_HUMAN	Immunoglobulin kappa constant
S4R460_HUMAN	Immunoglobulin heavy variable 3/OR16-9 (non-functional)
IGHM_HUMAN	Immunoglobulin heavy constant mu
IGHG3_HUMAN	Immunoglobulin heavy constant gamma 3
IGHG1_HUMAN	Immunoglobulin heavy constant gamma 1
IGHA2_HUMAN	Immunoglobulin heavy constant alpha 2
IGHA1_HUMAN	Immunoglobulin heavy constant alpha 1
IBP7_HUMAN	Insulin-like growth factor-binding protein 7
IBP6_HUMAN	Insulin-like growth factor-binding protein 6
IBP4_HUMAN	Insulin-like growth factor-binding protein 4
IBP2_HUMAN	Insulin-like growth factor-binding protein 2
PGBM_HUMAN	Basement membrane-specific heparan sulfate proteoglycan core protein
SAP3_HUMAN	Ganglioside GM2 activator
FIBG_HUMAN	Fibrinogen gamma chain
FIBB_HUMAN	Fibrinogen beta chain
FIBA_HUMAN	Fibrinogen alpha chain
FCGBP_HUMAN	IgGFc-binding protein
FA5_HUMAN	Coagulation factor V
CATD_HUMAN	Cathepsin D
CATB_HUMAN	Cathepsin B
CBPQ_HUMAN	Carboxypeptidase Q
CBPE_HUMAN	Carboxypeptidase E
CERU_HUMAN	Ceruloplasmin
CNTN2_HUMAN	Contactin-2
CLUS_HUMAN	Clusterin
CSTN3_HUMAN	Calsyntenin-3
CSTN1_HUMAN	Calsyntenin-1
SCG1_HUMAN	Secretogranin-1
CFAI_HUMAN	Complement factor I
CFAD_HUMAN	Complement factor D
CFAB_HUMAN	Complement factor B
CADH2_HUMAN	Cadherin-2
CD14_HUMAN	Monocyte differentiation antigen CD14
CADM2_HUMAN	Cell adhesion molecule 2
CA2D1_HUMAN	Voltage-dependent calcium channel subunit alpha-2/delta-1
CO9_HUMAN	Complement component C9
CO7_HUMAN	Complement component C7
CO6_HUMAN	Complement component C6

CO5_HUMAN	Complement C5
CO4A_HUMAN	Complement C4-A
CO2_HUMAN	Complement C2
C1R_HUMAN	Complement C1r subcomponent
C1QC_HUMAN	Complement C1q subcomponent subunit C
C1QB_HUMAN	Complement C1q subcomponent subunit B
B4GA1_HUMAN	Beta-1,4-glucuronyltransferase 1
B2MG_HUMAN	Beta-2-microglobulin
ZA2G_HUMAN	Zinc-alpha-2-glycoprotein
VAS1_HUMAN	V-type proton ATPase subunit S1
A4_HUMAN	Amyloid-beta A4 protein
APOC1_HUMAN	Apolipoprotein C-I
APOA4_HUMAN	Apolipoprotein A-IV
APOA2_HUMAN	Apolipoprotein A-II
APOA1_HUMAN	Apolipoprotein A-I
APLP2_HUMAN	Amyloid-like protein 2
APLP1_HUMAN	Amyloid-like protein 1
AMBP_HUMAN	Protein AMBP
ADA22_HUMAN	Disintegrin and metalloproteinase domain-containing protein 22
A2MG_HUMAN	Alpha-2-macroglobulin

Supplementary Table 2: Serum autoantibody findings and associated clinical characteristics in 10 patients with COVID-19.

Patient	Antibody	Syndrome
#1	NMDAR 1:100	Nystagmus, orofacial myoclonus (first present after resuscitation),<br>MRI:hypoxic brain damage
#2	Yo 1:100	Brachiofacial myclonia, EEG normal
#3	Caspr2 1:32	Unsteady gait and delir
#4	Myelin 1:100	Protracted waking after mechanical ventilation
#5	Myelin 1:100	Hyperactive delir
#6	Myelin 1:100	Delir
#7	Myelin 1:100	Delir
#8	Caspr2 1:10 and	Very severe delir

	NMDAR 1:10	
#9	Glycin 1:10	Mnestic difficulties (known AD)
#10	Glycin 1:10	Myalgia, delir

Supplementary Table 3. Change of the data matrix size after pre-processing steps.

Step	N features	M samples
Input data matrix size	5486	74
After sample removal	5486	72
After features with NA>35% removal	2230	72
After filtering out nonproteotypic peptides	1992	72
After peptides summarization	271	72
Final protein matrix size	271	72

Supplementary Table 4. Clinical groups used for analysis along with some demographic information. COVID-19 Patients were divided into cohort with elevated Procalcitonin levels (PCT>1) indicating a confounding superinfection (C19_high_PCT) and a cohort C19_low_PCT with normal Procalcitonin levels (PCT ≤ 1). All C19_high_PCT patients had WHO severity grade 4, C19_low_PCT patients on average had WHO severity grade 3. Two other groups were control and HSV Meningitis (HSVE).

	**Female**			**Male**			**Total**		
	**Avg Age**	**Avg PCT**	**NN pat**	**Avg Age**	**Avg PCT**	**NN pat**	**Avg Age**	**Avg PCT**	**NN pat**
C19_high_PCT	63.7	1.8	3	71.8	4.0	13	70.3	3.6	16
C19_low_PCT	69.2	0.1	5	68.8	0.3	17	68.9	0.2	22
control	57.6		17	58.8		11	58.1		28
HSVE	53.2		6				53.2		6
**Total**	**59.2**		**31**	**67.0**		**41**	**63.7**		**72**

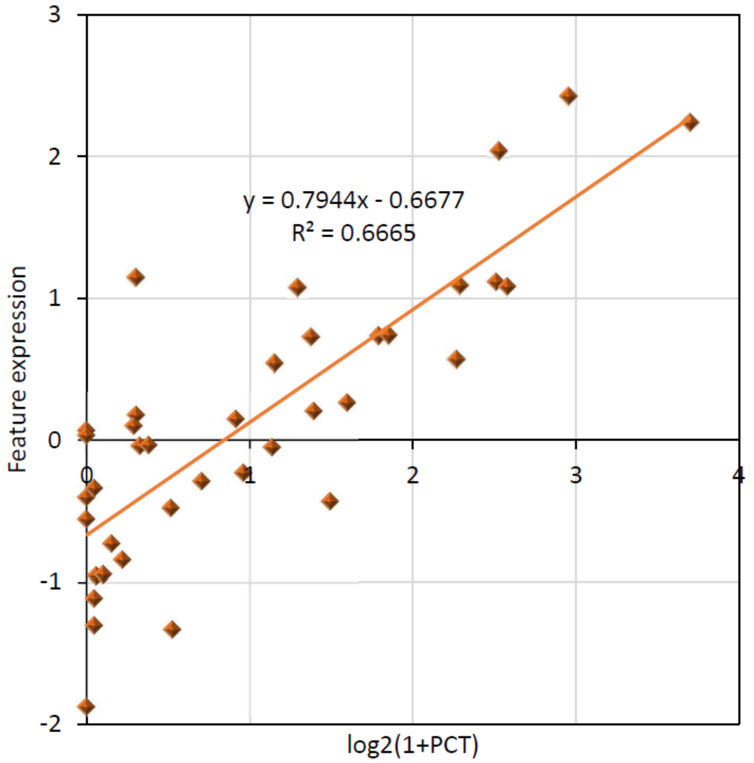

Supplementary Figure 1. Linear dependence of combined log2 expression of three proteins y= (C4A + CD14)/2 – NRCAM on transformed PCT level x = log2(1 + PCT). Proteins C4A and CD14 show increase of expression with increase of x and have nearly opposite dependence on sex and WHO grade, while NRCAM show decrease with increase of x and is almost independent on sex and WHO grade. Linear fit showed that approximately 67% of the variation in combined proteins expression can be explained by BSI ($R^2 = 0.67$). The fit was conducted on all COVID samples (as shown in the Figure) and on WHO grade 4 only samples with practically the same outcome.

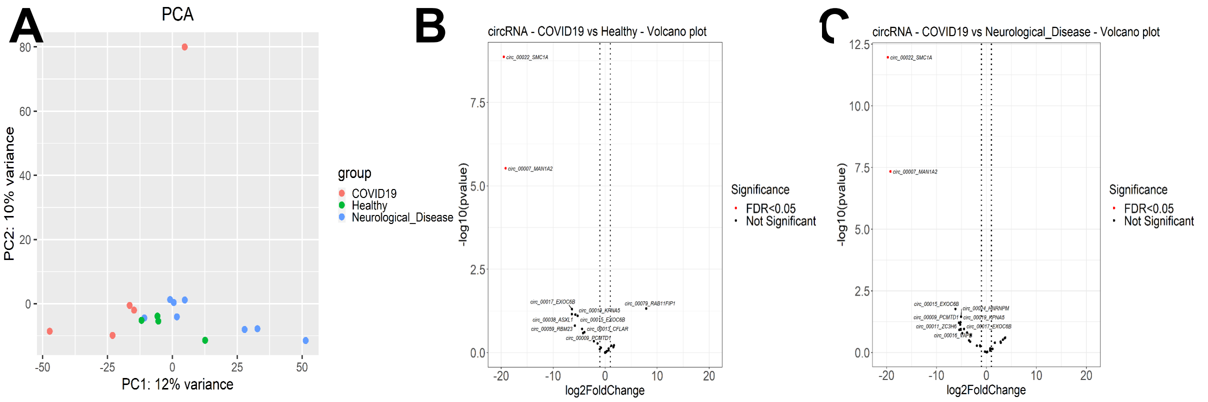

Supplementary Figure 2. CircRNA data analysis. (A) Principal component analysis (PCA) of circRNA profile in CSF of Covid-19 patients, healthy controls and patients with neurological disease. Differential expression analysis of circRNAs in (B) CSF of Covid-19 patients and healthy controls and (C) CSF of Covid-19 patients and patients with neurological disease. Red dots: FDR < 0.05.

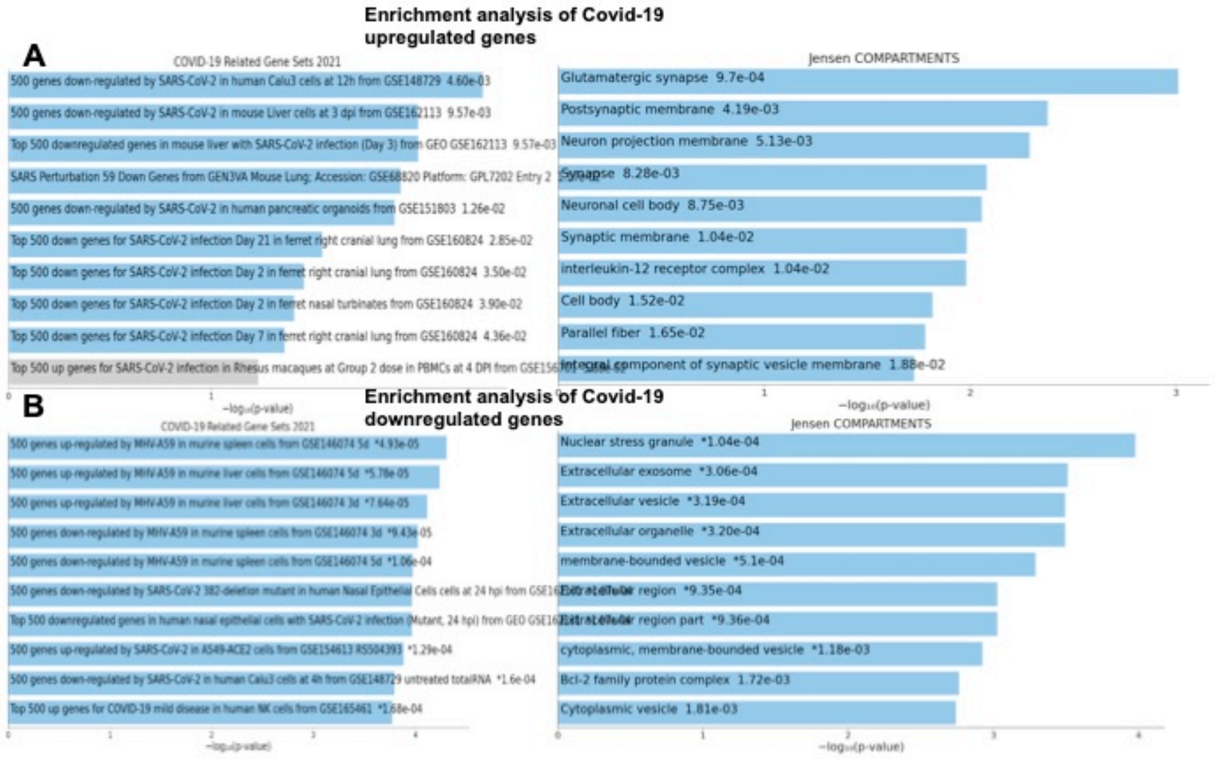

Supplementary Figure 3 Supplementary Figure. Enrichment analysis of deregulated genes in COVID-19 CSF. Top 10 enriched items in the COVID-19 related gene sets 2021 and cellular compartment localization in the analysis of (A) COVID-19 up-regulated genes and (B) COVID-19 down-regulated genes. The p-value is listed after each item; the items with p-value < 0.05 are significantly enriched; An asterisk (*) next to a p-value indicates the term also has a adjusted p-value <0.05.
